# Increased ERK signalling promotes inflammatory signalling in primary airway epithelial cells expressing Z α_1_-antitrypsin

**DOI:** 10.1093/hmg/ddt487

**Published:** 2013-10-04

**Authors:** Emily F.A. van ‘t Wout, Jennifer A. Dickens, Annemarie van Schadewijk, Imran Haq, Hang Fai Kwok, Adriana Ordóñez, Gillian Murphy, Jan Stolk, David A. Lomas, Pieter S. Hiemstra, Stefan J. Marciniak

**Affiliations:** 1 Department of Medicine, University of Cambridge, Cambridge Institute for Medical Research, Wellcome Trust/Medical Research Council Building, Hills Road, Cambridge CB2 0XY, United Kingdom,; 2 Department of Pulmonology, Leiden University Medical Center, Albinusdreef 2, Leiden 2333 ZA, the Netherlands,; 3 Department of Oncology, University of Cambridge, Robinson way, Cambridge CB2 0RE, United Kingdom,; 4 Proteases and Tumour Microenvironment Group, Cancer Research UK Cambridge Research Institute, Robinson way, Cambridge CB2 0RE, United Kingdom and; 5 Faculty of Medical Sciences, University College London, Maple House, Tottenham Court Road, London W1T 7NF, United Kingdom

## Abstract

Overexpression of Z α_1_-antitrypsin is known to induce polymer formation, prime the cells for endoplasmic reticulum stress and initiate nuclear factor kappa B (NF-κB) signalling. However, whether endogenous expression in primary bronchial epithelial cells has similar consequences remains unclear. Moreover, the mechanism of NF-κB activation has not yet been elucidated. Here, we report excessive NF-κB signalling in resting primary bronchial epithelial cells from ZZ patients compared with wild-type (MM) controls, and this appears to be mediated by mitogen-activated protein/extracellular signal-regulated kinase, EGF receptor and ADAM17 activity. Moreover, we show that rather than being a response to protein polymers, NF-κB signalling in airway-derived cells represents a loss of anti-inflammatory signalling by M α_1_-antitrypsin. Treatment of ZZ primary bronchial epithelial cells with purified plasma M α_1_-antitrypsin attenuates this inflammatory response, opening up new therapeutic options to modulate airway inflammation in the lung.

## INTRODUCTION

Alpha_1_-antitrypsin is a 52-kDa serine protease inhibitor (or serpin), primarily produced by hepatocytes, but also secreted locally by lung epithelial cells and alveolar macrophages (1,2). Its known function is to inhibit a number of serine proteases, including neutrophil elastase and proteinase 3, thereby preventing excessive degradation of the extracellular matrix. It has also been reported to exhibit anti-inflammatory properties, including the inhibition of tumor necrosis factorα (TNFα) gene expression (3), inhibition of a disintegrin and metalloprotease (ADAM)17 activity in neutrophils and endothelial cells (4,5) and the regulation of CD14 expression and cytokine release in monocytes (6,7).

The Z mutation (E342K) of α_1_-antitrypsin causes subtle misfolding of the protein that permits polymer formation and accumulation within the endoplasmic reticulum (ER) of hepatocytes or degradation by the proteasome leading to deficiency of the secreted protein (8,9). This causes hepatic cirrhosis through toxic gain-of-function within the liver, most likely due to the retention of polymers, and early-onset lung emphysema, due in large part to the loss of protease inhibition (10). The discovery of polymers in broncho-alveolar lavage fluid and pulmonary tissue (11,12), the pro-inflammatory nature of such extracellular polymers (11,13) and their identification many years after liver transplantation (14) led to the proposal that pulmonary pathology could be induced by polymer-induced toxic gain-of-function with inflammation as an additional mechanism (15).

Secreted proteins are first folded within the ER where quality control systems ensure that only properly folded proteins exit the organelle (16). Accumulation of unfolded or misfolded proteins within the ER induces ‘ER stress’, thereby activating intracellular signal transduction pathways, collectively called the unfolded protein response (UPR) (16). This complex cellular response evolved to restore ER homeostasis by reducing the load of newly synthesized protein while increasing the complement of molecular chaperones, which enhance ER protein-folding capacity, and increasing the efficiency of misfolded protein degradation (Endoplasmic reticulum-associated degradation, ERAD) (17,18). We have shown previously that mutant Z α_1_-antitrypsin is degraded predominantly by ERAD (19). Remarkably, the accumulation of polymers of Z α_1_-antitrypsin within the ER of hepatocytes does not activate the UPR but instead increases the cell's sensitivity to ER-stress upon a ‘second hit’ owing to impaired ER luminal protein mobility (20–22).

The transcription factor nuclear factor kappa B (NF-κB) regulates many genes involved in inflammation and cell death, including numerous cytokines and chemokines, e.g. interleukin (IL)-8 (23). Phosphorylation of NF-κB is classically mediated through the phosphorylation of inhibitor kappa-B alpha (IκBα); however, NF-κB can also be activated via mitogen-activated protein kinase (MAPK) signalling cascades (24,25).

Epidermal growth factor (EGF) and related mitogens such as heparin-binding EGF (HB-EGF), amphiregulin (AREG) and transforming growth factor (TGF)-α are synthesized as membrane-bound proteins that upon cleavage by metalloproteases (MPs), including ADAMs, bind to and activate the EGF receptor (EGFR) [reviewed in (26)]. Transactivation of the EGFR can also occur via activation of ADAMs by G-protein-coupled receptor signalling [reviewed in (27)]. Within the lung, EGFR activation can induce epithelial cell proliferation by activating extracellular signal-regulated kinases 1 and 2 (ERK1/2). This is mediated by Ras activation of c-Raf, causing phosphorylation of the mitogen-activated protein/extracellular signal-regulated kinase (MEK), which in turn phosphorylates ERK1/2 (28).

Mutants of members of the serpin superfamily, including α_1_-antitrypsin, have been shown to activate NF-κB signalling, postulated to be a response to the formation of protein polymers within the ER (20,21,29). However, this appears to be independent of their ability to prime cells for ER stress (29). Whether the local expression of Z α_1_-antitrypsin by airway epithelial cells *in vivo* leads to the formation of protein polymers and to the activation of the NF-κB pathway remains unclear. We report here the detection of NF-κB activation in primary bronchial epithelial cells isolated from patients homozygous for the Z mutation (ZZ) and demonstrate this to be mediated by increased ADAM17-dependent EGFR–MEK–ERK signalling in the absence of either detectable polymer formation or ER stress response. Instead, the activation of the EGFR in this setting represents a loss of M α_1_-antitrypsin phenotype.

## RESULTS

### Z α_1_-antitrypsin activates NF-κB in lung epithelial cells

**Figure 1.**
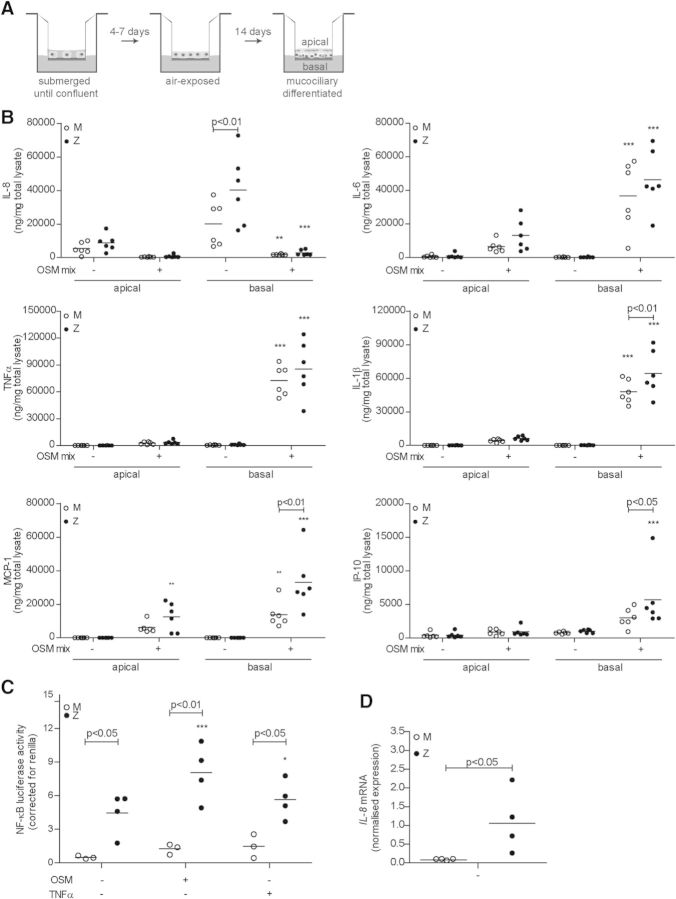
Z α_1_-antitrypsin expression enhances NF-κB signalling in lung epithelial cells. (**A**) Schematic diagram of culturing primary bronchial epithelial cells at an air (apical)–liquid (basal) interface. Once differentiated, epithelium is a pseudo-stratified cell layer composed of ciliated cells, goblet cells and basal cells. (**B**) Meso scale discovery^®^ of apically and basally secreted cytokines and chemokines (IL-8, IL-6, TNFα, IL-1β, MCP-1 and IP-10). Cells were treated with oncostatin M (100 ng/ml; OSM) and TNFα/IL1β (both 20 ng/ml; OSM-mix) as indicated for 48 h before harvesting apical washes and basal medium (mean, *n* = 6). (**C**) NF-κB luciferase activity of undifferentiated MM and ZZ cells. Submerged cells were cultured for 24 h and then transfected with luciferase reporters for 6 h and left 16 h with OSM or TNFα (20 ng/ml) as indicated. NF-κB reporter activity is corrected for *Renilla* (mean, *n* = 3–4). (**D**) Basal *IL-8* mRNA expression levels of undifferentiated primary bronchial epithelial cells measured by qPCR (mean, *n* = 4). *P < 0.05, **P < 0.01, ***P < 0.001 versus—with a two-way repeated-measurements ANOVA (Bonferroni *post hoc*).

It is well-recognized that overexpression of Z α_1_-antitrypsin activates the NF-κB response leading to pro-inflammatory cytokine release (20,21,30). We therefore asked whether expression of Z α_1_-antitrypsin regulated by its endogenous promoter in airway epithelial cells could also activate this pathway. Primary bronchial epithelial cells were differentiated into mucin-producing, ciliated epithelial cell layers (Fig. 1A), and a multiplex ELISA (Meso Scale Discovery^®^, Rockville, MD, USA) of apical washings (air exposed) and basal (liquid exposed) conditioned medium for IL-8, IL-6, TNFα, IL-1β, monocyte chemoattractant protein-1 (MCP-1) and interferon gamma-induced protein-10 (IP-10) was performed (Fig. 1B). This revealed that resting ZZ-differentiated primary bronchial epithelial cells secreted more IL-8 basally when compared with MM cells (*P* < 0.01). After combined stimulation with oncostatin M (OSM), TNFα and IL-1β (OSM-mix), the ZZ differentiated primary bronchial epithelial cells showed significantly higher release of MCP-1 (*P* < 0.01), IP-10 (*P* < 0.05) and IL-1β (*P* < 0.01) compared with MM controls. The reduced secretion of IL-8 most likely reflects the known inhibitory effect of OSM on IL-1β-induced IL-8 release (31). To determine whether this enhanced release of cytokines was mediated by increased NF-κB signalling, submerged cultures of patient-derived ZZ primary bronchial epithelial cells were induced to express increased levels of α_1_-antitrypsin by treatment with OSM and NF-κB activity was assayed using a luciferase reporter system (Fig. 1C) (29). Low levels of basal NF-κB luciferase activity were detected in MM primary bronchial epithelial cells, whereas ZZ cells showed significantly higher activity at baseline (*P* < 0.05 compared with MM; Fig. 1C). When α_1_-antitrypsin production was increased by treatment with OSM, the NF-κB activity in ZZ primary bronchial epithelial cells increased significantly (*P* < 0.001) and the difference between MM and ZZ cells increased still further (*P* < 0.01). Stimulation with TNFα, a cytokine known to induce NF-κB activation, showed the same difference between MM and ZZ cells (*P* < 0.05). The same effect was seen in Tet-On A549 cells overexpressing either M or Z α_1_-antitrypsin (Supplementary Material, Fig. S1). To test whether the baseline difference in NF-κB activity was related to the transfection of the reporter constructs, we measured transcription of the NF-κB-dependent chemokine IL-8, which confirmed basal levels of inflammatory signalling were higher in ZZ primary bronchial epithelial cells compared with controls (*P* < 0.05; Fig. 1D).

### Z α_1_-antitrypsin does not form detectable polymers nor causes ER stress in lung epithelial cells

**Figure 2.**
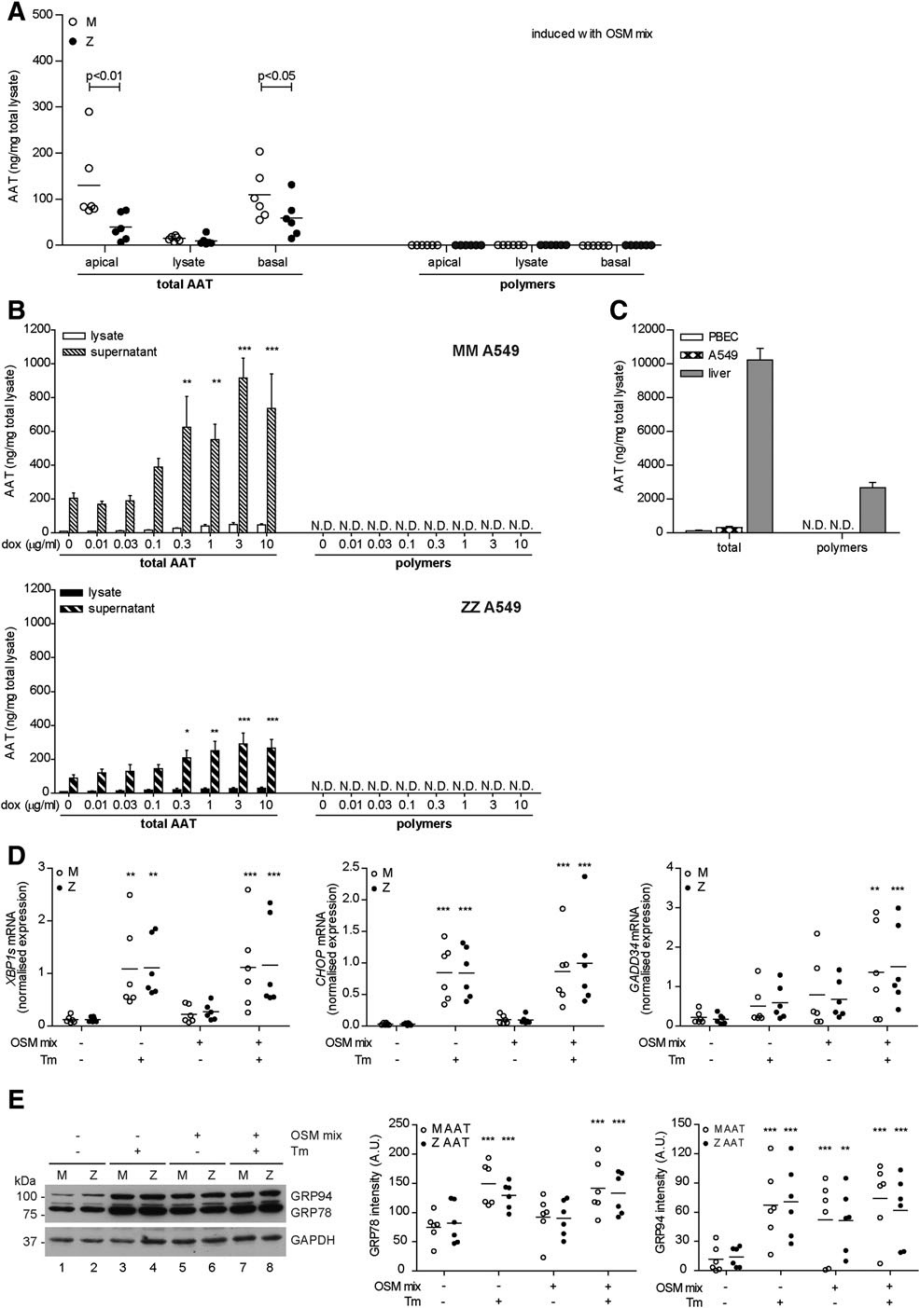
Polymer formation and an (exaggerated) ER stress response are not causing the augmented NF-κB response. (**A**) Total α_1_-antitrypsin (AAT) and α_1_-antitrypsin polymer production of fully differentiated primary bronchial epithelial cells stimulated with OSM-mix for 48 h. Note lack of polymer signal with the 2C1 antibody (mean, *n* = 6). (**B**) Total α_1_-antitrypsin and α_1_-antitrypsin polymer production of the overexpressing Tet-On A549 cells after inducing for 48 h with doxycycline (dox; mean ± SEM, *n* = 3). (**C**) Total α_1_-antitrypsin levels produced by ZZ lung epithelial cells compared with ZZ liver homogenate (*n* = 3 from one individual). (**D**) Quantitative RT-PCR of fully differentiated primary bronchial epithelial cells treated with OSM-mix for 48 h as indicated. Four hours before harvesting, cells were stimulated with tunicamycin (Tm; 1 µg/ml). *XBP1 splicing*, *CHOP* and *GADD34* mRNA levels are displayed normalized to the housekeeping genes RPL13A and ATP5B (mean, *n* = 6). (**E**) Western blot for GRP94 and GRP78 using anti-KDEL antibody. Cells were treated as in D but stimulated for 16 h with tunicamycin (mean, *n* = 6). N.D. not detectable. *P < 0.05, **P < 0.01, ***P < 0.001 versus—or 0 with a two-way repeated-measurements ANOVA (Bonferroni *post hoc*).

NF-κB activation by mutant serpins has previously been associated with the accumulation of protein polymers within the ER (20–22,29). This accumulation has also been shown to exaggerate ER stress upon a second hit (20,22). To verify whether this mechanism was responsible for the enhanced NF-κB signalling in fully differentiated ZZ primary bronchial epithelial cells, we first measured total secreted and intracellular α_1_-antitrypsin. Resting primary bronchial epithelial cells produced unquantifiable amounts of α_1_-antitrypsin, but after stimulation with OSM-mix for 48 h, α_1_-antitrypsin was detectable in both the apical washes and basal supernatant (Fig. 2A). Similar results were obtained after exclusion of the current smokers, indicating that the differences in smoking status of the patients between MM and ZZ patients from which cells were obtained did not explain the differences in production of α_1_-antitrypsin (data not shown). Using the 2C1 monoclonal antibody that specifically detects naturally occurring polymers of Z α_1_-antitrypsin (32), we found no evidence of polymer formation (Fig. 2A). Accordingly, we could detect no differences in ER protein mobility, which we have previously shown occurs in cells containing ER luminal polymers of α_1_-antitrypsin [(22) Supplementary Material, Fig. S2]. To determine whether the absence of polymer formation was a feature of lung epithelial cells, we generated stable transfected A549 lung carcinoma cell lines that conditionally expressed either M or Z α_1_-antitrypsin under the control of a Tet-On responsive promoter. As expected, M α_1_-antitrypsin-expressing A549 cells secreted five-times more α_1_-antitrypsin than did Z α_1_-antitrypsin-expressing A549 cells (Fig. 2B). Again, we were unable to detect protein polymers in either the supernatant or cellular lysates (Fig. 2B). As polymer formation is dependent upon α_1_-antitrypsin concentration (8), we compared the relative levels of α_1_-antitrypsin in tissue from an explanted cirrhotic ZZ liver (Fig. 2C) with those in cultured airway epithelial cells. This revealed a 100-fold higher level of α_1_-antitrypsin in hepatic tissue and significant polymer accumulation (Fig. 2C). While polymerization of α_1_-antitrypsin *in vitro* is highly dependent upon protein concentration, the concentration dependence of polymerization within the crowded environment of the ER *in vivo* is not known. Therefore, to determine the critical concentration for the polymerization of Z α_1_-antitrypsin in living cells, we induced the expression of Z α_1_-antitrypsin in Tet-On stable CHO stable cells (22) and measured both total α_1_-antitrypsin and polymer levels (Supplementary Material, Fig. S3A). This revealed that levels of 300 ng α_1_-antitrypsin per 1 mg of total lysate protein are necessary before intracellular polymers can be detected in these cells (Supplementary Material, Fig. S3A). To test this finding in lung epithelial-derived cells, we induced expression of Z α_1_-antitrypsin in Tet-On A549 cells with doxycycline and augmented the protein level by inhibiting ERAD with lactacystin, a selective proteasome inhibitor. This increased the concentration of intracellular Z α_1_-antitrypsin of >300 ng α_1_-antitrypsin per 1 mg of total lysate, whereupon polymers were detected (Supplementary Material, Fig. S3B). It therefore seems likely that the low levels of α_1_-antitrypsin produced by airway epithelia are insufficient to generate detectable polymers.

To define whether Z α_1_-antitrypsin expressed in epithelial cells alters the ER stress response, we induced expression of α_1_-antitrypsin in fully differentiated primary bronchial epithelial cells with OSM-mix in the presence or absence of the ER stress-inducing toxin tunicamycin. We detected no differences in the basal or OSM-mix-stimulated levels of *spliced X-box binding protein-1* (*XBP1s*), *CCAAT-enhancer-binding protein homologous protein* (*CHOP*) and *growth arrest and DNA damage-inducible gene 34* (*GADD34*) mRNA between MM and ZZ primary bronchial epithelial cells (Fig. 2D). As expected, tunicamycin increased the level of these transcripts; however, there was not an exaggerated ER stress response in ZZ epithelial cells (Fig. 2D). Similarly, we were unable to detect differences in ER stress by western blot for the KDEL-positive chaperones, glucose-regulated protein94 (GRP94) and GRP78, in ZZ and MM cells (Fig. 2E).

### Loss of M α_1_-antitrypsin leads to increased activation of ERK, which is dependent on MEK and EGFR

**Figure 3.**
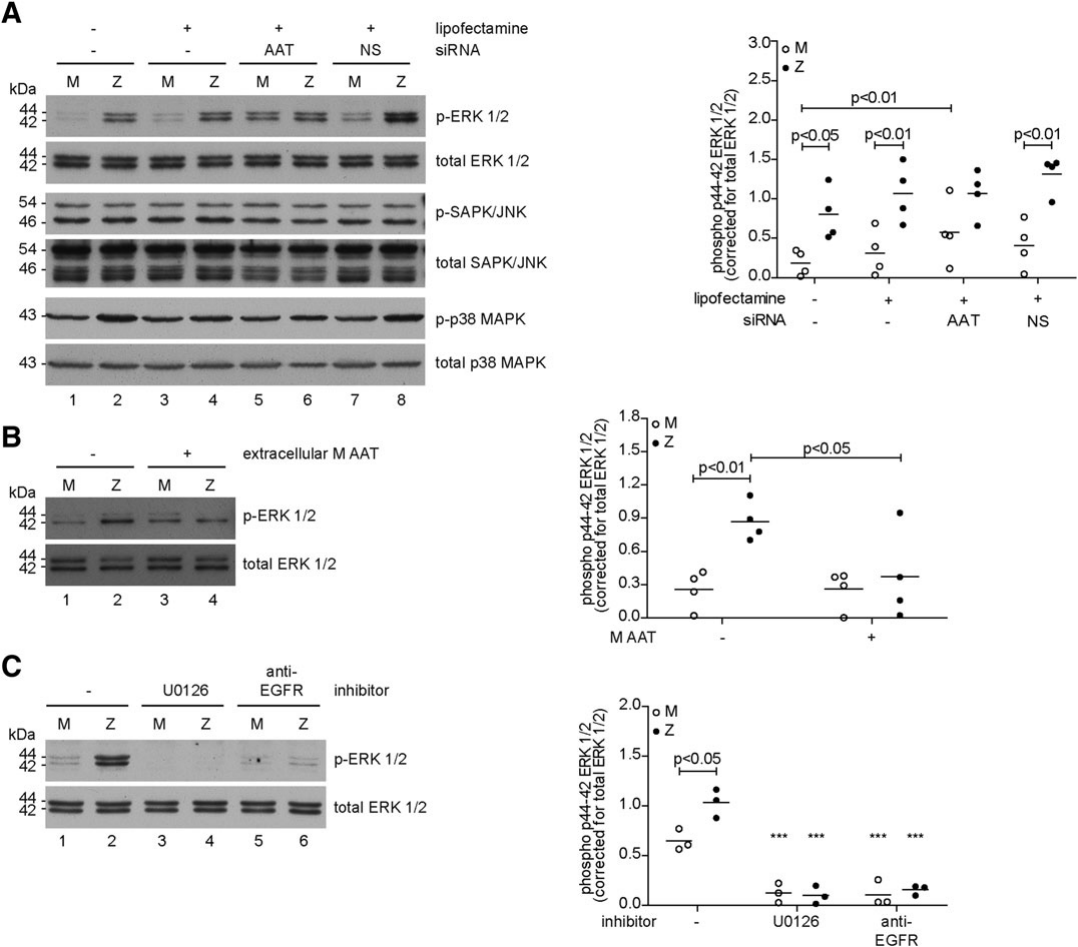
Increased NF-κB response in ZZ primary bronchial epithelial cells is dependent on the ERK/MEK/EGFR pathway. (**A**) Representative western blot of the activation of the MAP kinases ERK1/2, JNK and p38 MAPK of whole cell lysates from undifferentiated primary bronchial epithelial cells knocked-out for AAT with siRNA. Cells were cultured overnight and transfected for 24 h and left 48 h before harvesting. Neuroserpin (NS) siRNA served as a control. Densitometry of four independent experiments in duplicate (mean, *n* = 4). (**B**) ZZ primary bronchial epithelial cells treated for 24 h with 1 mg/ml purified plasma M α_1_-antitrypsin normalized ERK1/2 levels. Densitometry of four independent experiments in duplicate (mean, *n* = 4). (**C**) ZZ primary bronchial epithelial cells treated with 10 µM U0126 (a specific MEK inhibitor) for 8 h or 2 µg/ml anti-EGFR blocking antibody for 24 h. Densitometry of three independent experiments in duplicate (mean, *n* = 3). *P < 0.05, **P < 0.01, ***P < 0.001 versus—or 0 with a two-way repeated-measurements ANOVA (Bonferroni *post hoc*).

In order to understand the mechanism of inflammatory signalling in ZZ epithelial cells, we next evaluated activation of the NF-κB pathway components, inhibitor of nuclear factor kappa-B kinase subunit beta (IKKβ), IκBα and p65. To evaluate MAPK signalling, we also measured JNK, p38 MAPK and ERK1/2. This revealed a significant difference only in the activation of ERK (*P* < 0.05; Fig. 3A and Supplementary Material, Fig. S4). Interestingly, depletion of α_1_-antitrypsin by siRNA caused phosphorylation of ERK in MM primary bronchial epithelial cells, but did not alter the phosphorylation of ERK in ZZ cells (Fig. 3A). This effect was specific for ablation of α_1_-antitrypsin, because silencing a non-specific serpin, neuroserpin (NS), did not increase phosphorylation of ERK in MM cells. This suggested that it was the lack of (M) α_1_-antitrypsin, rather than the presence of Z α_1_-antitrypsin, that might be responsible for the phosphorylation of ERK in ZZ primary bronchial epithelial cells. To test this, we treated ZZ primary bronchial epithelial cells with plasma-purified M α_1_-antitrypsin and observed a suppression of ERK phosphorylation (*P* < 0.05; Fig. 3B). We wished to determine whether this loss of function phenotype reflected a lack of anti-inflammatory activity in Z α_1_-antitrypsin or if cells secreted insufficient Z α_1_-antitrypsin. As concentration of plasma-purified Z α_1_-antitrypsin to a degree required for this experiment would result in its polymerization, we instead transiently transfected HeLa cells with either M or Z α_1_-antitrypsin or empty vector as control. After transfection, the cells produced high levels of α_1_-antitrypsin, with ZZ cells producing ∼20% of the amount that MM cells produced (444 ng/mg α_1_-antitrypsin in the total lysate versus 1995 ng/mg α_1_-antitrypsin in the total lysate respectively; Supplementary Material, Fig. S5A). Although 14% (33 ng/mg total lysate) of the extracellular Z α_1_-antitrypsin formed polymers (data not shown), the protein was able to inhibit phosphorylation of ERK1/2 to a similar degree as M α_1_-antitrypsin (Supplementary Material, Fig. S5B). This result is consistent with a model in which ZZ primary bronchial epithelial cells secrete insufficient α_1_-antitrypsin to inhibit ERK1/2 phosphorylation, rather than Z α_1_-antitrypsin lacking the anti-inflammatory activity *per se*.

To determine the mechanism of ERK phosphorylation in ZZ primary bronchial epithelial cells, we treated cells with U0126, a specific inhibitor of MEK, or an anti-EGFR blocking monoclonal antibody. Both reagents abrogated the phosphorylation of ERK, indicating that the EGFR–MEK-signalling pathway was involved and was activated by the ligand-binding site of EGFR (Fig. 3C). Although under some circumstances, the activation of ERK1/2 can lead to epithelial cell proliferation, we did not observe differences in the rate of MM and ZZ cell proliferation (Supplementary Material, Fig. S6A). Attempts to assess the effect of ERK1/2 inhibition in each cell type were hampered by toxicity, but it did not appear that modulation of ERK1/2 activation affected proliferation of MM or ZZ cells differentially. Treatment of ZZ cells with purified M α_1_-antitrypsin did not appear to affect the rate of proliferation (Supplementary Material, Fig. S6B).

### ZZ primary bronchial epithelial cells generate higher levels of ADAM17-dependent EGFR ligands

**Figure 4.**
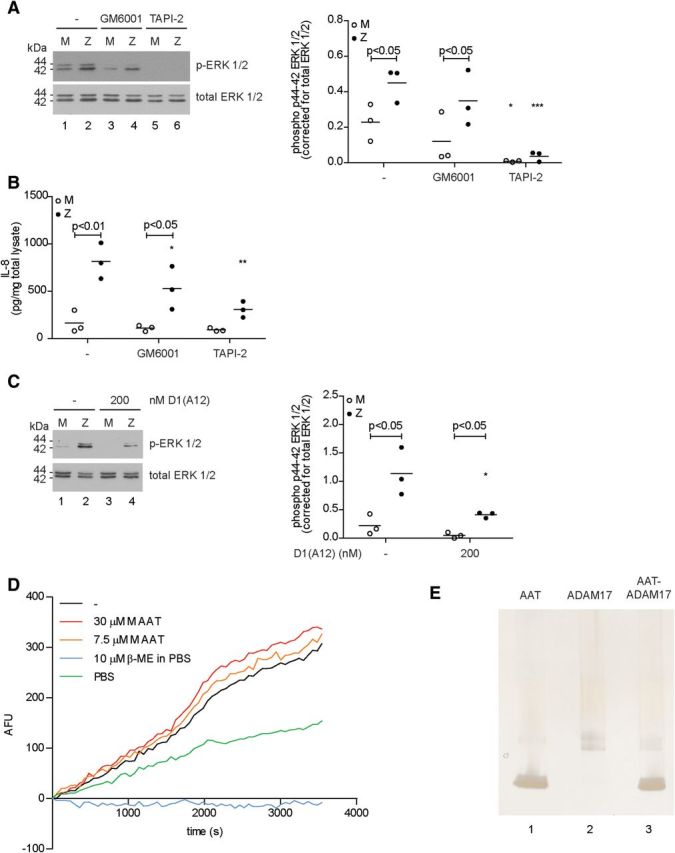
M α_1_-antitrypsin protects primary bronchial epithelial cells against constitutively activated ERK1/2 in an ADAM17-dependent fashion. (**A**) Western blot for phosphorylated ERK1/2 of whole cell lysates from primary bronchial epithelial cells treated with 25 µM GM6001 (MP inhibitor) or 25 µM TAPI-2 (MMP and ADAM17 inhibitor) for 24 h. Densitometry of three independent experiments in duplicate (mean, *n* = 3). (**B**) IL-8 secretion in supernatant of primary bronchial epithelial cells treated as in A (mean, *n* = 3). (**C**) Treatment with D1(A12), a specific ADAM17 blocking antibody, inhibits phosphorylation of ERK1/2 in ZZ cells after 6 h. (–) represents a control IgG. Densitometry of three independent experiments in duplicate (mean, *n* = 3). (**D**) ADAM17 (1 nM) was incubated with 7.5 or 30 µM plasma M α_1_-antitrypsin. ADAM17 activity was assayed against a fluorogenic substrate. β-mercaptoethanol (10 µM) in PBS was used as a positive control for inhibition. (–) represents baseline ADAM17 activity against the fluorogenic substrate. (**E**) Silver stain of a Bis–Tris non-reducing PAGE revealed no binding of ADAM17 to plasma M α_1_-antitrypsin incubated in a 1:1 molar ratio for 1 h at 37°C. *P < 0.05, **P < 0.01, ***P < 0.001 versus—with a two-way repeated-measurements ANOVA (Bonferroni *post hoc*).

We reasoned that this increased EGFR signalling might reflect increased cleavage of membrane-tethered ligands by MMPs and/or ADAMs. Therefore, we incubated MM and ZZ primary bronchial epithelial cells with GM6001, a broad-spectrum metalloproteinase (MP) inhibitor, or TAPI-2, a broad-spectrum MP inhibitor with enhanced ADAM17 inhibitory activity. TAPI-2 completely blocked the phosphorylation of ERK1/2 in ZZ cells (*P* < 0.001), whereas GM6001 failed to affect phosphorylation (Fig. 4A). Furthermore, only treatment with TAPI-2 reduced secretion of IL-8 from ZZ cells (*P* < 0.01) (Fig. 4B). As this suggested the involvement of ADAM17, we next tested the effect of a specific ADAM17 blocking antibody, D1(A12) (33). At 200 nM, a concentration known to block the activity of ADAM17 (34), we observed a substantial decrease in phosphorylation of ERK1/2 (*P* < 0.05; Fig. 4C).

A previous report suggested that M α_1_-antitrypsin can directly inhibit ADAM17 derived from neutrophils (4), and so we attempted to reproduce this observation. We first performed an *in vitro* ADAM17 activity assay by incubating 1 nM ADAM17 with 0–30 µM purified plasma M α_1_-antitrypsin but were unable to detect any inhibition of ADAM17 (Fig. 4D). When using 10 µM β-mercaptoethanol in PBS or PBS alone, two potent ADAM17 inhibitors, we were able to inhibit its activity, confirming the functionality of our assay (Fig. 4D). Moreover, we were unable to detect the formation of a complex between ADAM17 and either form of α_1_-antitrypsin (Fig. 4E). Although it remains controversial as to whether cytoplasmic phosphorylation of ADAM17 plays an important regulatory role (35), we also tested for phosphorylation of ADAM17 in MM and ZZ cells grown with or without supplementary M α_1_-antitrypsin (Supplementary Material, Fig. S7). We detected no differences between these conditions.

**Figure 5.**
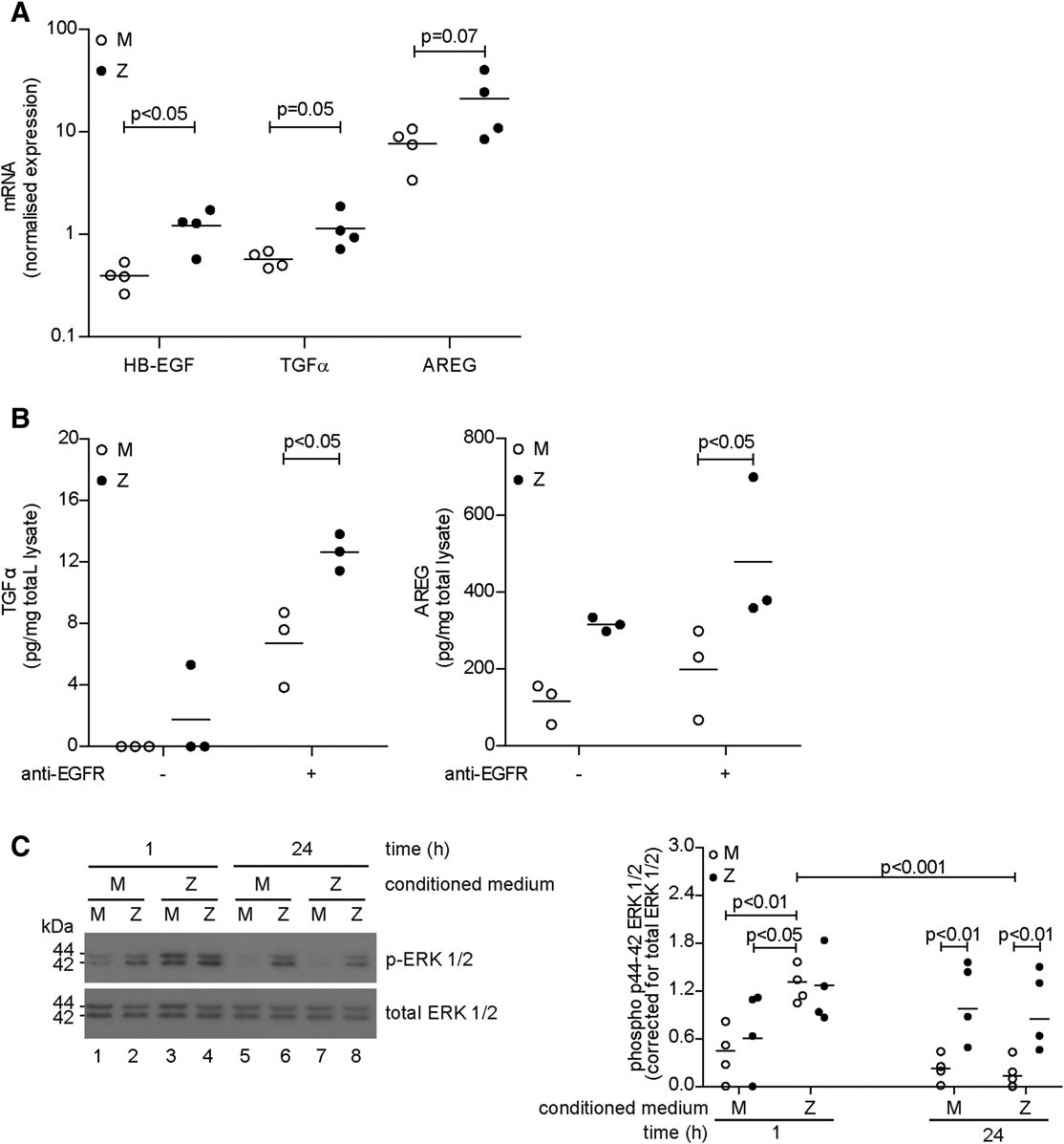
EGFR ligands are enriched in ZZ primary bronchial epithelial cells. (**A**) Basal expression levels of the EGFR ligands HB-EGF, TGFα and AREG in undifferentiated primary bronchial epithelial cells, measured by qPCR. EGF was not quantifiable (mean, *n* = 4). (**B**) Increased TGFα and AREG in the cell supernatant of ZZ primary bronchial epithelial cells after blockade of the EGFR, quantified by ELISA. HB-EGF was undetectable (mean, *n* = 3). (**C**) Conditioned medium from ZZ primary bronchial epithelial cell culture given to MM primary bronchial epithelial cells and vice versa. Phosphorylation of ERK1/2 was measured 1 and 24 h after media exchange. Densitometry of four independent experiments in duplicate (mean, *n* = 4). *P < 0.05, **P < 0.01, ***P < 0.001 versus—with a two-way repeated-measurements ANOVA (Bonferroni *post hoc*).

To explore this pathway further, we next measured whether ZZ primary bronchial epithelial cells generated more EGFR ligands than controls. We detected significantly higher levels of mRNA encoding HB-EGF and TGFα in ZZ cells compared with MM controls (*P* < 0.05 and *P* = 0.05; Fig. 5A). When the EGFR was blocked using a monoclonal antibody to prevent ligand binding to its receptor, significantly more released TGFα and AREG was detected in ZZ cells compared with MM cells (*P* < 0.05; Fig. 5B). Consistent with these observations, when we treated MM primary bronchial epithelial cells with conditioned medium derived from ZZ cultures, we observed a significant increase in phosphorylation of ERK1/2 after 1 h (*P* < 0.01), which returned to baseline after 24 h (*P* < 0.001; Fig. 5C).

**Figure 6.**
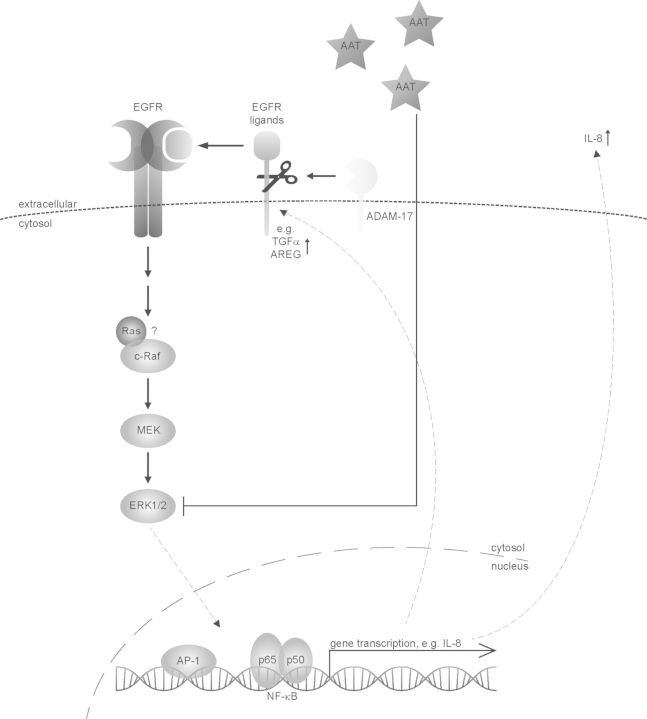
ZZ primary bronchial epithelial cells show an enhanced inflammatory response dependent of the ERK/EGFR/ADAM17 pathway. Impaired expression of α_1_-antitrypsin in primary bronchial epithelial cells leads to increased phosphorylation of ERK1/2, which is dependent on MEK, EGFR and ADAM17. M α_1_-antitrypsin modulates this inflammatory response via a yet undefined mechanism.

Taken together, these data indicate that the increased NF-κB signalling in ZZ primary bronchial epithelial cells is caused by phosphorylation of ERK1/2. This is due to increased availability of ADAM17-dependent EGFR ligands leading to activation of the EGFR and signalling via MEK (Fig. 6). Surprisingly, we were unable to detect the formation of polymers of α_1_-antitrypsin in ZZ primary bronchial epithelial cells or A549 lung adenocarcinoma cells overexpressing the protein, which may reflect the low levels of α_1_-antitrypsin expression of which these cells are capable.

## DISCUSSION

For many years, it was thought that an imbalance between protease and antiprotease activity was solely responsible for the accelerated onset of emphysema in patients homozygous for the Z allele of α_1_-antitrypsin [reviewed in (36)]. However, α_1_-antitrypsin has been found to possess additional roles to its antiprotease activity, including a range of anti-inflammatory properties (3–5), and the Z alleles can increase inflammatory NF-κB signalling when overexpressed pulmonary epithelial cells (20,21,30). The results of our current study using primary bronchial epithelial cells in which α_1_-antitrypsin is expressed under the control of its endogenous promoter confirm and extend these findings. We found that at the low levels of α_1_-antitrypsin expression that occur in these cells, clinically relevant polymer formation is unlikely to occur. Sufficient α_1_-antitrypsin is generated by wild-type cells to suppress ERK1/2 and NF-κB signalling, but this anti-inflammatory effect appears to be lost in ZZ epithelia, suggesting a novel mechanism for airway pathology in α_1_-antitrypsin deficiency. Surprisingly, while ADAM17 is required to generate secreted EGFR ligands mediating inflammatory signals in these ZZ cells, we were unable to detect a direct inhibition of ADAM17 by M α_1_-antitrypsin.

Many heterologous overexpression systems have reported the presence of Z α_1_-antitrypsin polymers both intracellular and in conditioned medium (20,21). Unexpectedly, but importantly, we were unable to detect 2C1-positive polymers in primary bronchial epithelial cultures. Although the 2C1 monoclonal antibody is highly specific for polymers, it is formally possible that it is less sensitive than other anti-polymer antibodies, for example, ATZ11. However, in our hands both have similar avidity towards Z polymers, but the polyclonal antibody ATZ11 is less specific for polymers, detecting both Z monomers as well as Z polymers (32). Even if very low levels of Z α_1_-antitrypsin polymer are made within bronchial epithelial cells, it is unlikely to affect cellular function as we were unable to detect impaired ER protein mobility nor altered ER stress responsiveness as we have done previously for polymer-expressing cells (22).

We were unable to identify all components required for ERK1/2 activation in ZZ primary bronchial epithelial cells. It is likely there exists a significant level of redundancy in this system as we were able to detect multiple EGFR ligands to be increased in the conditioned medium of ZZ cultures. In our system, α_1_-antitrypsin did not directly inhibit ADAM17 as has been reported for neutrophils (4). Interestingly, it has recently been speculated that endocytosed α_1_-antitrypsin may modulate ADAM17 in endothelial cells (5). If intracellular α_1_-antitrypsin can indeed modulate these pathways, multiple potential mechanisms may explain this effect. ADAM-dependent transactivation of the EGFR by activation of G-protein-coupled receptors can also occur [reviewed in (27)]. For example, IL-8 can induce EGFR phosphorylation in epithelial cells via its receptors C-X-C chemokine receptor1 (CXCR1) and CXCR2 (28,37), while binding of α_1_-antitrypsin to IL-8 has been reported to prevent activation of CXCR1 (4). Although there are contradictory reports concerning the expression of CXCR1 and CXCR2 on bronchial epithelial cells (38,39), we found that IL-8 release is increased in ZZ primary bronchial epithelial cells, which potentially might provide an autocrine inflammatory signal in the absence of sufficient α_1_-antitrypsin. Alternatively, C-C chemokine ligand20 (also known as MIP-3α) and its receptor C-C chemokine receptor6 have been shown to activate ADAM17 causing transactivation of EGFR (40,41).

Taken together, our experiments have demonstrated that airway epithelial cells have anti-inflammatory activity owing to the local synthesis of M α_1_-antitrypsin from its endogenous promoter and that this effect is lacking in ZZ homozygous epithelial cells because of a lack of α_1_-antitrypsin secretion. This raises the possibility that α_1_-antitrypsin augmentation may have unanticipated effects within the airway. It also raises the potential that targeted anti-EGFR therapy might have anti-inflammatory effects within the lung.

## MATERIALS AND METHODS

### Reagents and antibodies

OSM (100 ng/ml) was purchased from R&D systems, Minneapolis, MN, USA, and TNFα and IL-1β (both 10 ng/ml) from Peprotech, Rocky Hill, NJ, USA. Tunicamycin (1 µg/ml) was bought from Sigma–Aldrich, St. Louis, MO, USA, U0126 (10 nM) from Promega, Madison, WI, USA, monoclonal (Ab-3) anti-EGFR antibody (2 µg/ml), GM6001 (25 µM) and TAPI-2 (25 µM) all from Calbiochem, Darmstadt, Germany and D1(A12) (200 nM) (33). Purified plasma M α_1_-antitrypsin (1 mg/ml) was derived as described previously (42). All antibodies for immunoblotting were purchased from Cell Signaling Technology, Danvers, MA, USA, except secondary HRP-labelled antibodies (Sigma–Aldrich) and GRP94 and GRP78 were detected using an anti-KDEL monoclonal antibody (StressGen).

### Cell cultures

Primary bronchial epithelial cells were cultured submerged in a 1:1 mixture of Dulbecco's modified Eagle's medium (DMEM) and bronchial epithelial growth medium (BEGM; Clonectics, San Diego, CA, USA) with BEGM SingleQuot supplements and growth factors (Clonectics), 1.5 µg/ml bovine serum albumin (BSA; Sigma–Aldrich), 1 mM HEPES (Invitrogen, Life Technologies, Breda, the Netherlands) and 100 U/ml penicillin and 100 µg/ml streptomycin (Sigma–Aldrich), shortened as full medium, at 37°C, 5% CO_2_ (43). Starvation medium consists of full medium except BSA and the SingleQuot supplements EGF and BPE.

We carried out siRNA-mediated knockdown of α_1_-antitrypsin using a SMARTpool of ONTARGETplus α_1_-antitrypsin siRNA (Dharmacon, Lafayette, CO, USA) or SERPIN1 siRNA (Dharmacon) as a mock control. In general, 10 nM siRNA and 1 µl RNAiMAX (Invitrogen) were used according to manufacturer's descriptions, and α_1_-antitrypsin expression, measured with qPCR and ELISA, was knocked down >90% after 72 h by this method (Supplementary Material, Fig. S8).

Primary bronchial epithelial cells were differentiated as described previously (44). Briefly, cells were cultured submerged until confluence on semi-permeable Transwell membranes (Corning Costar, Cambridge, MA, USA) in B/D medium with addition of retinoic acid (end-concentration 15 ng/ml; Sigma–Aldrich) and subsequently cultured air-exposed for 14 days to allow mucociliary differentiation.

A549 cells were obtained from American Type Culture Collection (ATCC) and stably transfected with the pTetON vector (Clontech) to obtain Tet-On A549 cells. ELISA confirmed that production of endogenous α_1_-antitrypsin protein was below the level of detection in these cells (data not shown). These were stably transfected with pTRE2-hyg plasmid encoding M or Z α_1_-antitrypsin as described previously (42). Cells were maintained in DMEM with 10% (v/v) tetracycline-free foetal bovine serum, 100 U/ml penicillin, 100 µg/ml streptomycin, 400 µg/ml geneticin and 400 µg/ml hygromycin B (selective antibiotics from Invitrogen) at 37°C, 5% CO_2_. Expression of α_1_-antitrypsin was usually induced using 2 µg/ml doxycycline (Sigma–Aldrich) for 48 h. HeLa cells were obtained from ATCC and transient transfected with pcDNA3.1 encoding M or Z α_1_-antitrypsin (or empty vector) as shown previously (19).

### Patient groups

PiZZ α_1_-antitrypsin-deficient patients were recruited in the Leiden University Medical Center (LUMC; Leiden, the Netherlands). ZZ primary bronchial epithelial cells were acquired by bronchial biopsy, with approval from the Medical Ethical Committee of the LUMC. Briefly, bronchial biopsies were washed with PBS, divided in 2-mm pieces and placed into a fibronectin/collagen-coated 24-well plate. Twice daily, the explants were fed with 20 µl B/D medium until they became adherent (maximum of three days). Then, primary bronchial epithelial cells were expanded submerged with 500 µl B/D medium, replaced triweekly. When cultures reached confulence cells were frozen down in liquid nitrogen until further use. MM primary bronchial epithelial cells were obtained from tumour-free resected lung tissue as described previously (43) and matched to ZZ primary bronchial epithelial cells according to sex, GOLD-stage (0-III) and smoking status (non-, ex- or current smoker) (Supplementary Material, Table S1). In each case, care was taken to ensure that cells were sourced only from the 2nd–3rd branches of the bronchial tree in order that results from each were directly comparable.

### Measurement of total α_1_-antitrypsin, polymerized α_1_-antitrypsin and cytokine release

Total and polymerized α_1_-antitrypsin were measured in whole cell lysate (intracellular) and supernatant (secreted) by ELISA as described previously (32). Cytokine release of fully differentiated cells was measured using a 4-plex Meso Scale Discovery kit (IL-6, IL-8, TNFα and IL-1β) and singleplex kits (MCP-1 and IP-10; Meso Scale Discovery^®^). EGFR ligands were measured using commercially available ELISAs following manufacturer's protocol (R&D systems). IL-8 release in supernatant of submerged primary bronchial epithelial cultures was quantified using an IL-8 ELISA kit (Sanquin, Amsterdam, the Netherlands).

### Protein mobility assay

Submerged cultured primary bronchial epithelial cells were grown overnight on coated 35-mm glass-bottom petri dishes (MatTek Corporation, Ashland, MA, USA) and transiently transfected with an ER-GFP plasmid (45). Live cells were imaged on an LSM510 confocal microscope (DuoScan; Carl Zeiss Inc., Thornwood, NY, USA) at 37°C as defined in Ordonez *et al.* (22). Briefly, ER-GFP was visualized with an x63/1.4NA oil objective at 488-nm laser and fluorescence recovery after photobleaching experiments were performed. Fluorescence recovery curves were obtained by transforming ﬂuorescence intensities into a percentage scale in which the pre-bleach time point represents 100% of ﬂuorescence intensity.

### Luciferase activity assays

Transfection with luciferase reporter plasmids was typically performed in 6-well plates with 1 µg of either p(5x)ATF6-luc (Firefly) or pELAM1-luc (Firefly) and 50 ng of pRL-TK (*Renilla*) as a transfection efficacy control (29). Cells were transfected for 6 h with 2 µl Lipofectamine LTX (Invitrogen) in serum- and antibiotic-free OptiMEM according to manufacturer's instructions and lysed the next day using the recommended protocol of the Dual-Luciferase Reporter Assay (Promega).

### Western blotting

Cells were lysed in 50 µl buffer H [10 mm HEPES, pH 7.9, 50 mm NaCl, 500 mm sucrose, 0.1 mm EDTA and 0.5%, (v/v) Triton X-100, 1 mm PMSF, 1× Complete™ protease inhibitor cocktail (Roche Applied Science, Mannheim, Germany) supplemented with phosphatase inhibitors [10 mm tetrasodium pyrophosphate, 17.5 mm β-glycerophosphate and 100 mm NaF; (46,47)]. Samples were run on a 10% SDS–PAGE gel and transferred onto a nitrocellulose membrane. After blocking with PBS/0.05% Tween-20 (v/v)/5% skim-milk (w/v), the membrane was incubated with the primary antibody (1:1000) in TBS/0.05% Tween-20 (v/v)/5% BSA (w/v) overnight at 4°C. The HRP-labelled antibody was incubated for 1 h in blocking buffer and developed with ECL (ThermoScientific).

### Quantitative RT-PCR

Total RNA was isolated and normalized mRNA levels were calculated using RPL13A and ATP5B as housekeeping genes (48). Primers used are described in Supplementary Material, Table S2 (49,50). IQ SYBRGreen supermix (Bio-rad, Herculus, CA, USA) was used for amplification of the cDNA.

### ADAM17 activity and binding assay

ADAM17 activity assay was performed as descried earlier (51). In brief, 1 nM purified recombinant ADAM17 was incubated with or without 30–0 µM purified plasma M α_1_-antitrypsin and assayed for ADAM17 activity using the fluorogenic substrate MOCAc-Lys-Pro-Leu-Gly-Leu-Dap(Dnp)-Ala-Arg-NH_2_ (R&D systems). Purified plasma M α_1_-antitrypsin (0.5 µg) was incubated for 1 h at 37°C in a 1:1 molar ratio with ADAM17, ran on a Native Page Bis–Tris (3–12% w/v; Invitrogen) gel and visualized by Silver stain. Both ADAM17 and purified plasma M α_1_-antitrypsin were incubated in 50 mm Tris–HCl (pH 7.4), 100 mm NaCl and 10 mm CaCl.

### Statistical analysis

Results from primary bronchial epithelial cells are expressed as single patients (each dot is the average of one patient in duplicate). Results from Tet-On A549 are shown as mean ± SEM. Data were analysed using two-way repeated-measures analysis of variance (ANOVA) or Student t-test as appropriate. Differences with *P*-values < 0.05 were considered to be statistically significant.

## SUPPLEMENTARY MATERIAL

Supplementary Material is available at *HMG* online.

## FUNDING

This work was supported by the Medical Research Council UK (G1002610 to S.J.M.), the Netherlands Asthma Foundation (grant no. 3.2.08.0032 to E.F.A.W. and P.S.H.) and the Medical Research Council (UK) and NIHR UCLH Biomedical Research Centre (both to D.A.L.).

## Supplementary Material

Supplementary Data
