# Supplementary material for: Increased ERK signalling promotes inflammatory signalling in primary airway epithelial cells expressing Z α1-antitrypsin
Source: Hum Mol Genet. 2013 Oct 4;23(4):929–41. doi: 10.1093/hmg/ddt487 (PMC4007119; doi:10.1093/hmg/ddt487)
Supplement: Supplementary Data [file supp_23_4_929__index.html]

Increased ERK signalling promotes inflammatory signalling in primary airway epithelial cells expressing Z α1-antitrypsin — Increased ERK signalling promotes inflammatory signalling in primary airway epithelial cells expressing Z α1-antitrypsin — Supplementary Data 

# Increased ERK signalling promotes inflammatory signalling in primary airway epithelial cells expressing Z α1-antitrypsin

## Supplementary Data

Supplementary Data

**Files in this Data Supplement:**

- Supplementary Data - Docx file
- Supplementary Figure 1 - tif file
- Supplementary Figure 2 - tif file
- Supplementary Figure 3 - tif file
- Supplementary Figure 4 - tif file
- Supplementary Figure 5 - tif file
- Supplementary Figure 6 - tif file
- Supplementary Figure 7 - tif file
- Supplementary Figure 8 - tif file
