# Supplementary material for: Increased ERK signalling promotes inflammatory signalling in primary airway epithelial cells expressing Z α1-antitrypsin
Source: Hum Mol Genet. 2013 Oct 4;23(4):929–41. doi: 10.1093/hmg/ddt487 (PMC4007119; doi:10.1093/hmg/ddt487)
Supplement: Supplementary Data [file supp_ddt487_ddt487supp.docx]

**Supplementary data**

**Figure legends**

**Figure S1. Tet-On A549 cells overexpressing Z α_1_-antitrypsin show enhanced NF-κB signalling upon an inflammatory stimulus.** Cells were treated as in Fig.1*C*, only cells were induced with doxycycline (dox) instead of OSM (mean ± SEM, n=3).

**Figure S2. Z α_1_-antitrypsin** **does not alter ER protein mobility.** Primary bronchial epithelial cells were transiently transfected with ER-GFP and fluorescence recovery was measured by fluorescence recovery after photo-bleaching (FRAP). Recovery is displayed relative to pre-bleach fluorescence intensity (mean ± SEM, n=9-10).

**Figure S3. Critical concentration for polymer formation within cells.** (A) Dose-response doxycycline on a CHO stable cell line overexpressing Z α_1_-antitrypsin under the control of a Tet-On responsive promotor. Intracellular total and polymeric Z α_1_-antitrypsin levels are measured by ELISA. (B) Z α_1_-antitrypsin-expressing A549 cells induced with doxycycline and stimulated in the presence of lactacystin. Intracellular total and polymeric Z α_1_-antitrypsin levels are measured by ELISA.

**Figure S4. Increased NF-κB response in ZZ primary bronchial epithelial cells is caused by increased phosphorylated ERK.** (A) Representative western blots of the activation of the NF-κB proteins IKKβ and NF-κB p65 and degradation of total IκBα of whole cell lysates from undifferentiated primary bronchial epithelial cells knocked-down for α_1_-antitrypsin by siRNA (see Fig.3*A*). Densitometry was done on four independent experiments in duplicate (mean, n=3). (B) Densitometry of the Western blots shown in Fig.3*A* (mean, n=3-4).

**Figure S5. Overexpression of Z α_1_-antitrypsin downregulates ERK1/2 phosphorylation.** (A) α_1_-antitrypsin production after transfection of HeLa cells with pcDNA3.1 containing M or Z α_1_-antitrypsin (or empty vector as control). (B) Representative western blot of phospho ERK1/2 in HeLa cells after transfection with M or Z α_1_-antitrypsin. High amounts of Z α_1_-antitrypsin are able to inhibit phospho ERK1/2 at the same rate as M α_1_-antitrypsin does.

**Figure S6. Increased ERK1/2 phosphorylation in ZZ cells does not enhance proliferation.** (A) Cells were seeded at 3^.^10^4^ cells/well and cultured for 48 hours as indicated. Starvation medium is full medium except BSA and the SingleQuot supplements EGF and BPE to minimise the effects of exogenous growth factors. FR180204, a specific ERK inhibitor did not change proliferation rates. U0126, a specific MEK inhibitor, was toxic to both MM and ZZ cells. (B) ZZ cells were seeded at 3^.^10^4^ cells/well and cultured for 48 hours in the presence of 1mg/ml M α_1_-antitrypsin.

**Figure S7. ZZ cells do not display increased phosphorylation of ADAM17.** (A) ZZ primary bronchial epithelial cells were treated for 24 hours with 1 mg/ml purified plasma M α_1_-antitrypsin. Representative western blot of phosphorylated ADAM17 (Thr735) (n=3).

**Figure S8. Confirmation of α_1_-antitrypsin** **knock-down by siRNA in primary bronchial epithelial cells.** (A) *α_1_-antitrypsin* mRNA knock-down was >90% efficient (left panel) and after α_1_-antitrypsin up-regulation with OSM mix >98% efficient (right panel). Neuroserpin (NS) siRNA served as a control (mean, n=3). (B) α_1_-antitrypsin was undetectable in cell supernatant after knock-down of α_1_-antitrypsin, measured by ELISA (mean, n=3).

**Table S1. Patient characteristics.**

**Table S2. qPCR primers.**

**Tables**

**Table S1. Patient characteristics.**

|  | PiMM controls | PiZZ patients |
| --- | --- | --- |
| age (mean, range) | 61 (51-83) | 52 (43-57) |
| sex (M/F) | 3/3 | 3/3 |
| GOLD-stage (0/I/II/III) | 2/2/1/1 | 3/1/1/1 |
| smoking status (current/never/ex) | 2/0/4 | 0/1/5 |

**Table S2. qPCR primers.**

| Name | Forward primer | Reverse primer | Melting  temp. (°C) | Ref. |
| --- | --- | --- | --- | --- |
| AAT | 5’ AAG GCA AAT GGG AGA GAC CC 3’ | 5’ AAGAAGATGGCGGTGGCAT 3’ | 60 | ([50](#_ENREF_50)) |
| AREG | 5’ GGT GGT GCT GTC GCT CTT G 3’ | 5’ AGG TGT CAT TGA GGT CCA ATC C 3’ | 62 | - |
| CHOP | 5′ GCA CCT CCC AGA GCC CTC ACT CTC C 3′ | 5′ GTC TAC TCC AAG CCT TCC CCC TGC G 3′ | 62 | ([48](#_ENREF_48)) |
| EGF | 5’ TGC AGA GGG ATA CGC CCT AA 3’ | 5’ CAA GAG TAC AGC CAT GAT TCC AAA 3’ | 62 | - |
| GADD34 | 5' ATG TAT GGT GAG CGA GAG GC 3' | 5’ GCA GTG TCC TTA TCA GAA GGC 3' | 62 | ([51](#_ENREF_51)) |
| HB-EGF | 5’ TGG ACC TTT TGA GAG TCA CTT TAT CC 3’ | 5’ CGT GCT CCT CCT TGT TTG GT 3’ | 62 | - |
| IL-8 | 5’ CTG GAC CCC AAG GAA AAC 3’ | 5’ TGG CAA CCC TAC AAC AGA C 3’ | 60 | - |
| TGFα | 5’ AGG TCC GAA AAC ACT GTG AGT 3’ | 5’ AGC AAG CGG TTC TTC CCT TC 3’ | 62 | - |
| XBP1s | 5′ TGC TGA GTC CGC AGC AGG TG 3′ | 5′ GCT GGC AGG CTC TGG GGA AG 3′ | 62 | ([48](#_ENREF_48)) |
