# Supplementary figures and images for: Increased ERK signalling promotes inflammatory signalling in primary airway epithelial cells expressing Z α1-antitrypsin
Source: Hum Mol Genet. 2013 Oct 4;23(4):929–41. doi: 10.1093/hmg/ddt487 (PMC4007119; doi:10.1093/hmg/ddt487)

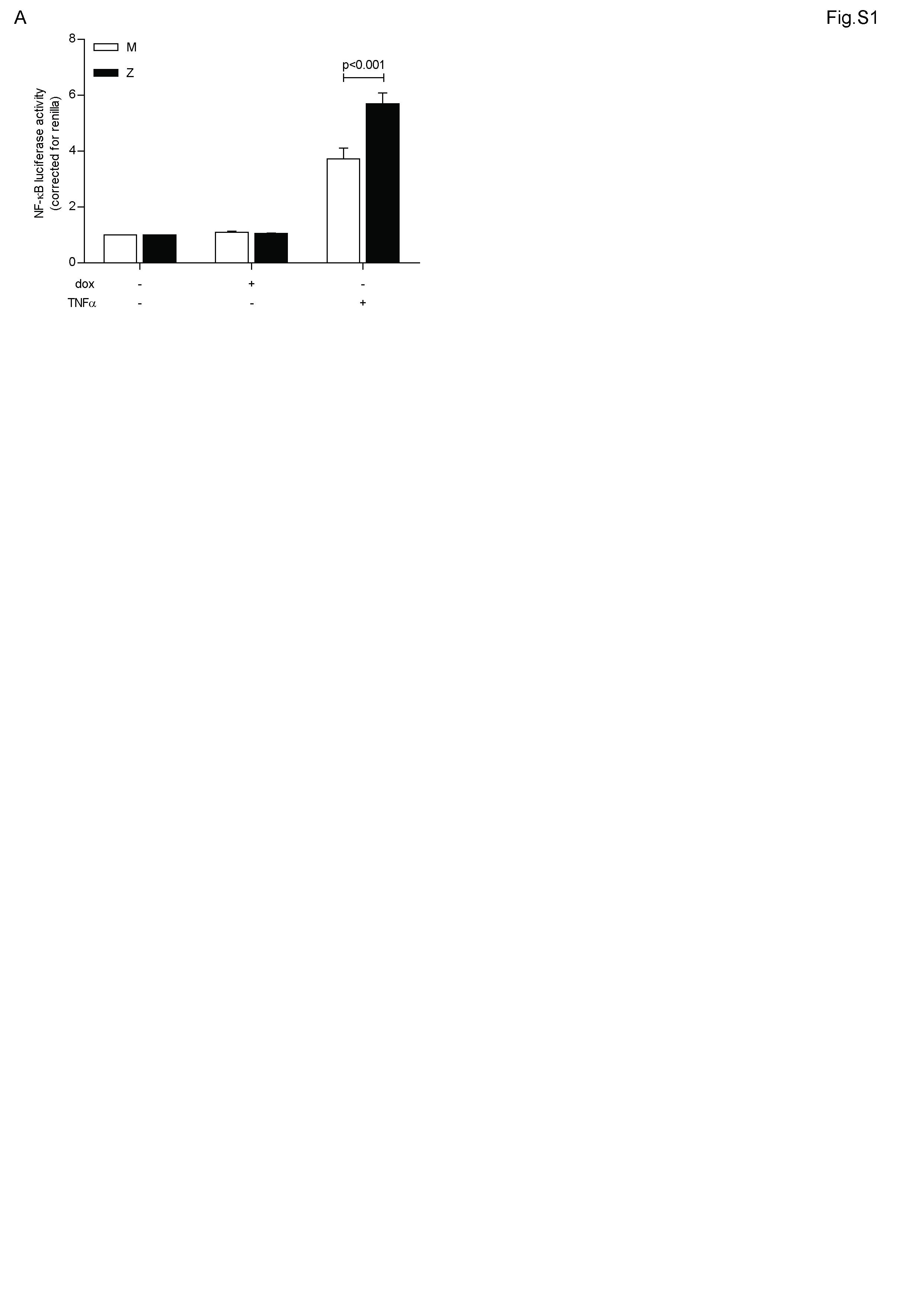

Supplement: Supplementary Data [file supp_ddt487_ddt487supp_fig1.tif]

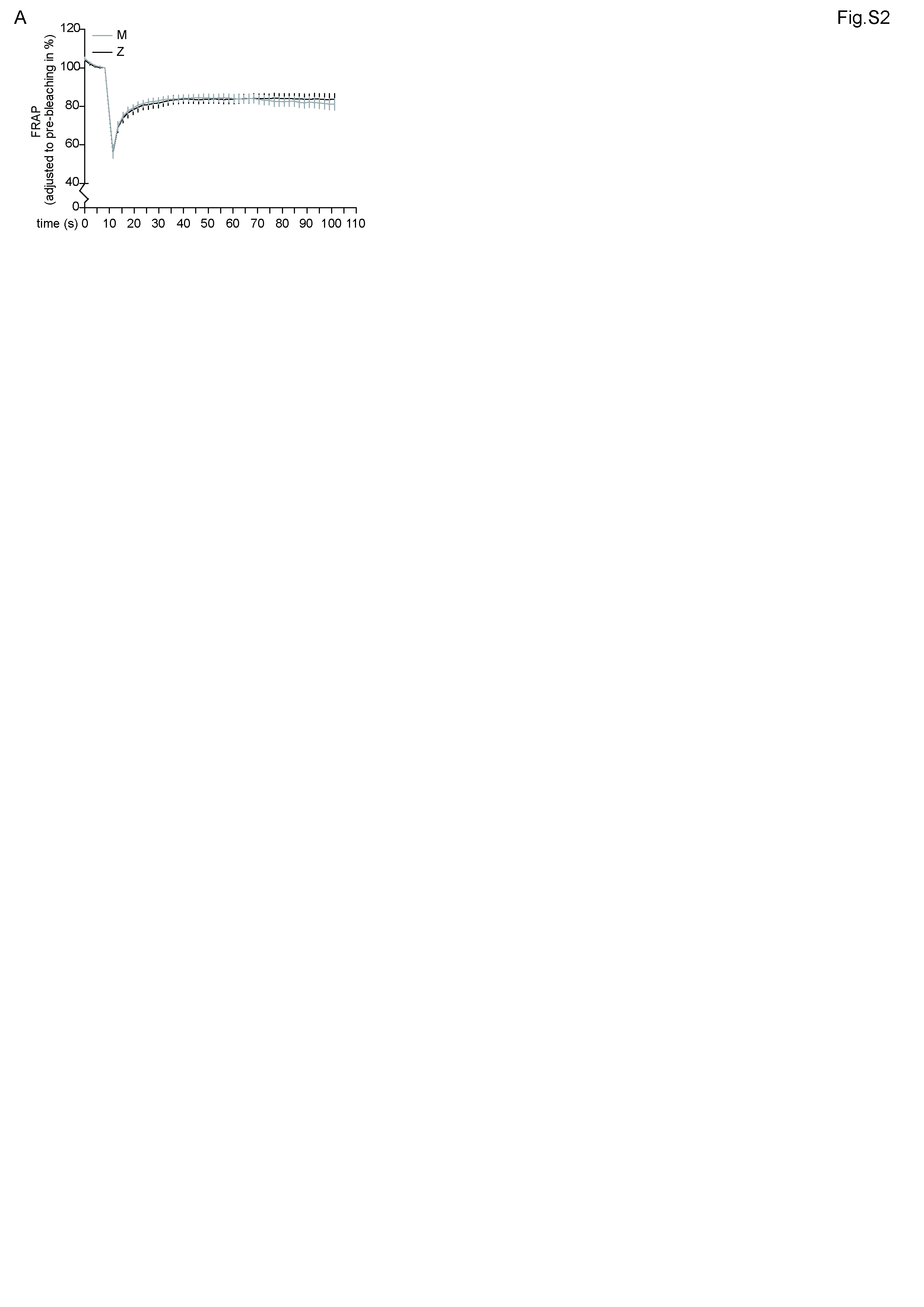

Supplement: Supplementary Data [file supp_ddt487_ddt487supp_fig2.tif]

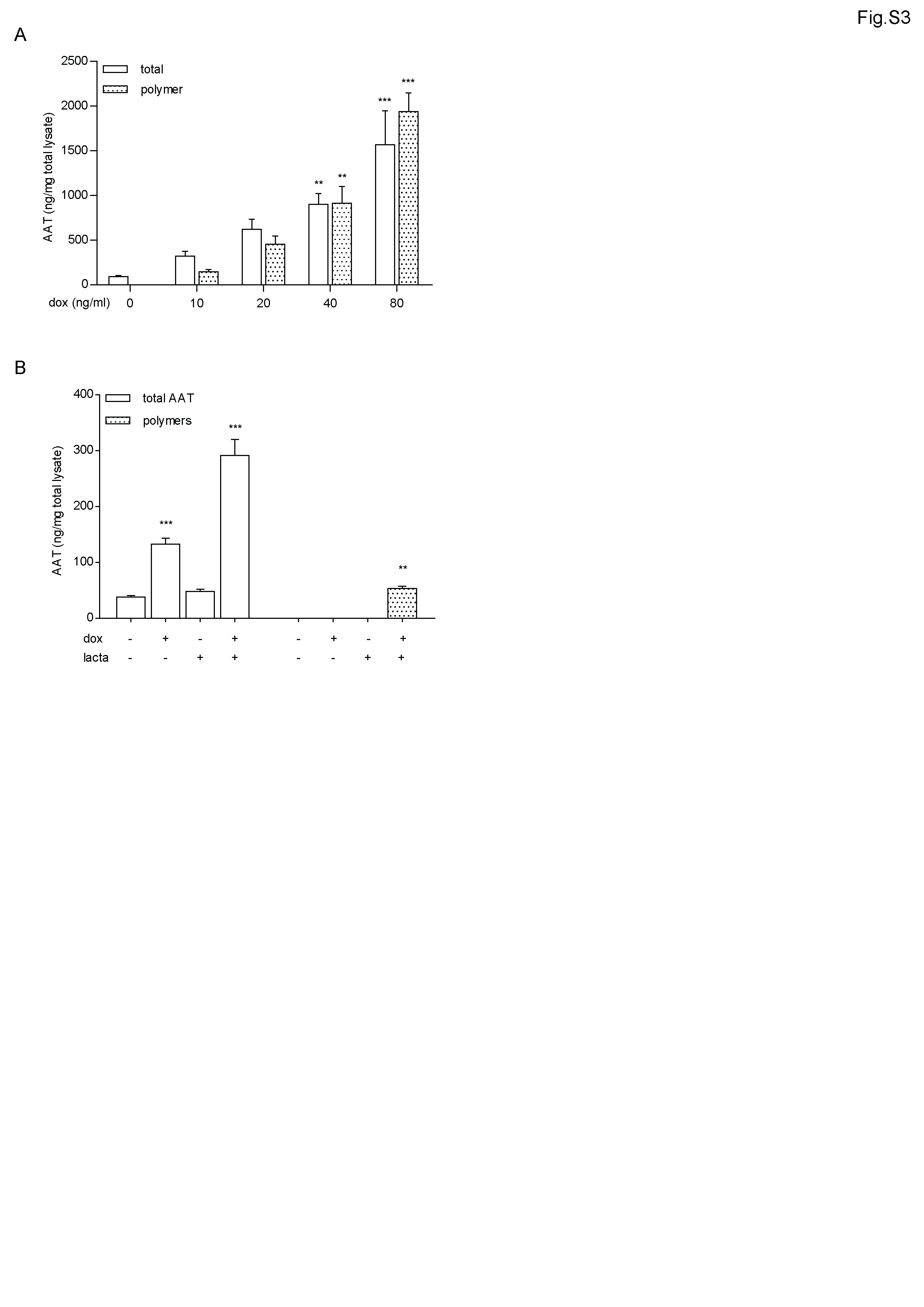

Supplement: Supplementary Data [file supp_ddt487_ddt487supp_fig3.tif]

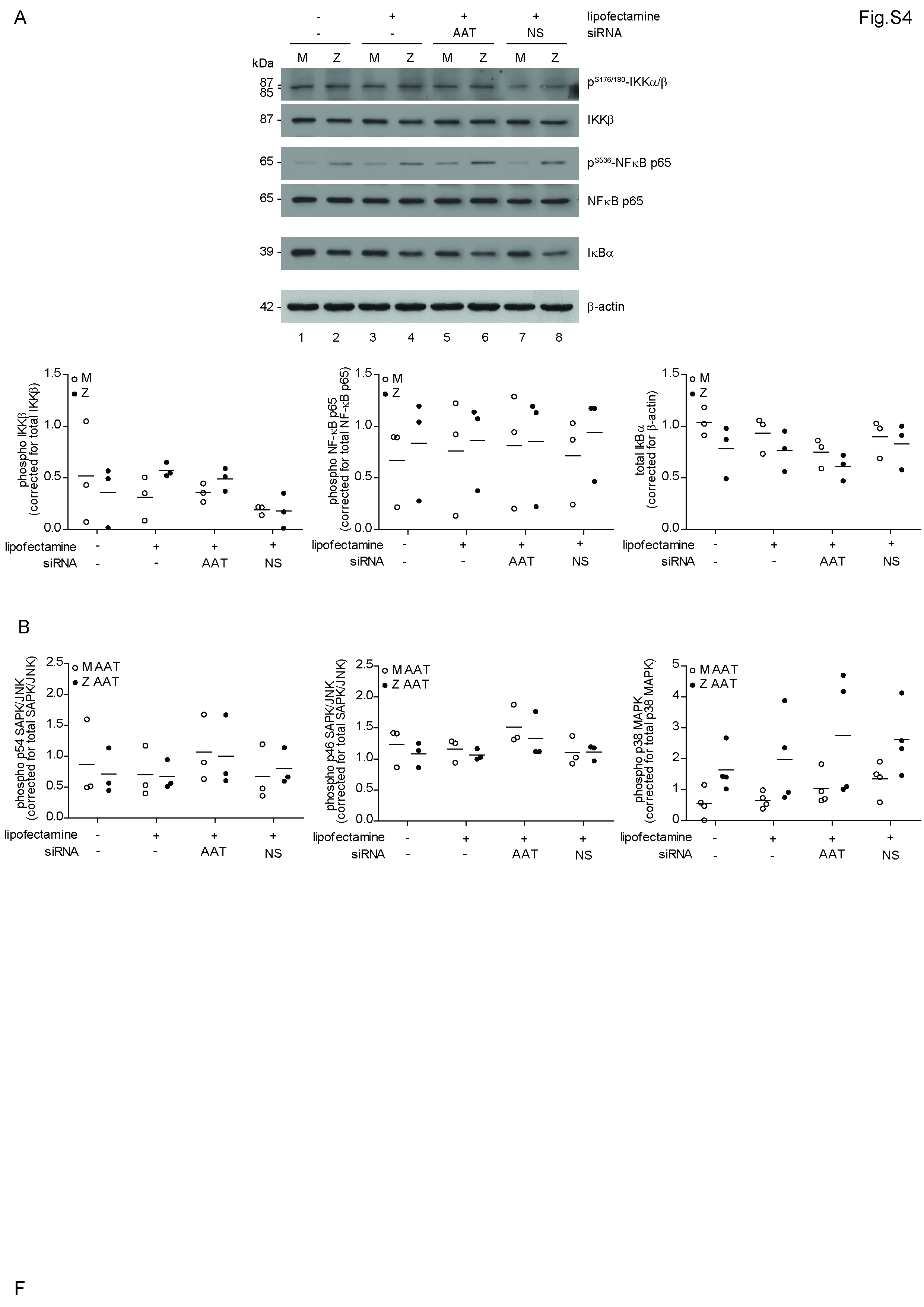

Supplement: Supplementary Data [file supp_ddt487_ddt487supp_fig4.tif]

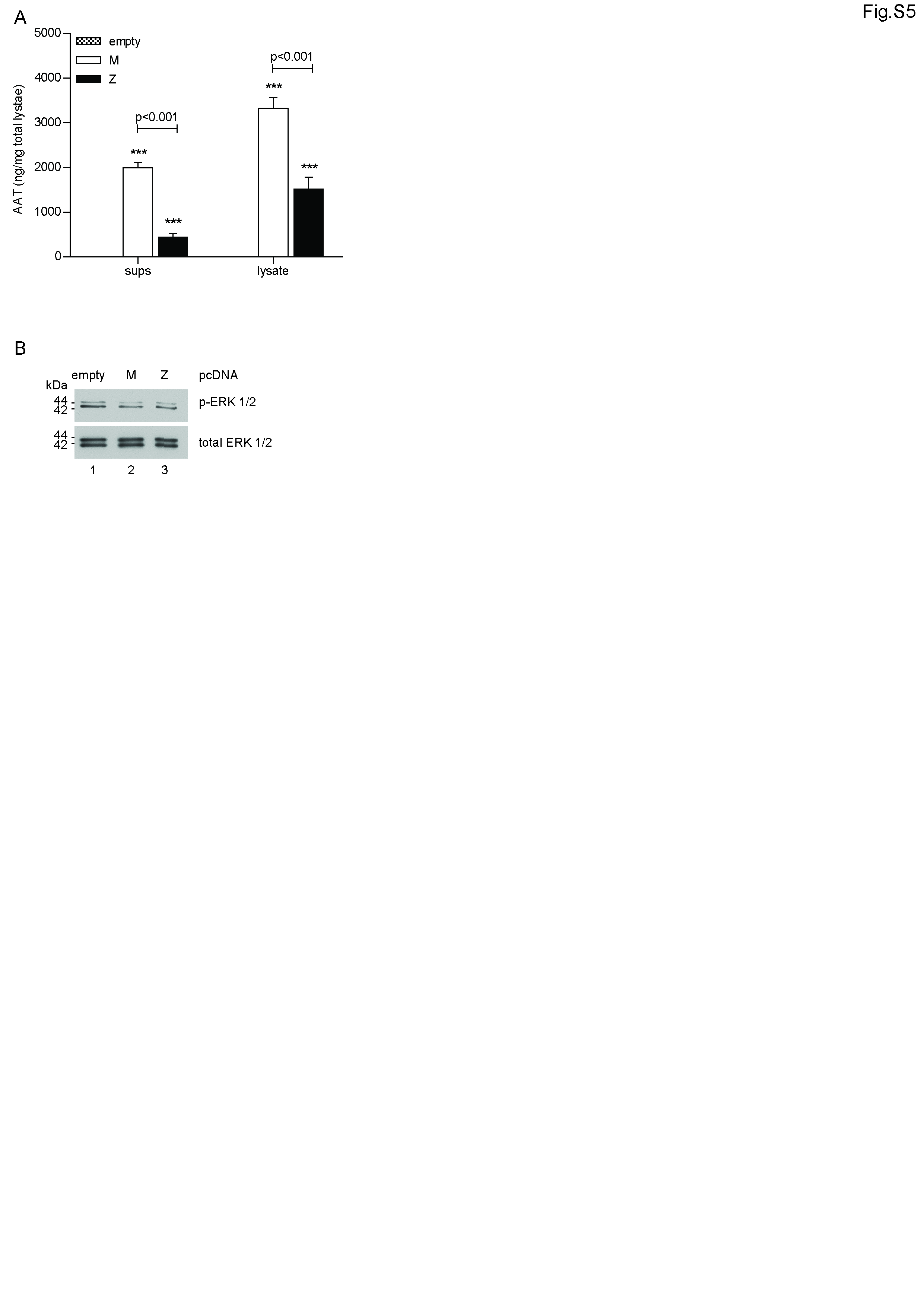

Supplement: Supplementary Data [file supp_ddt487_ddt487supp_fig5.tif]

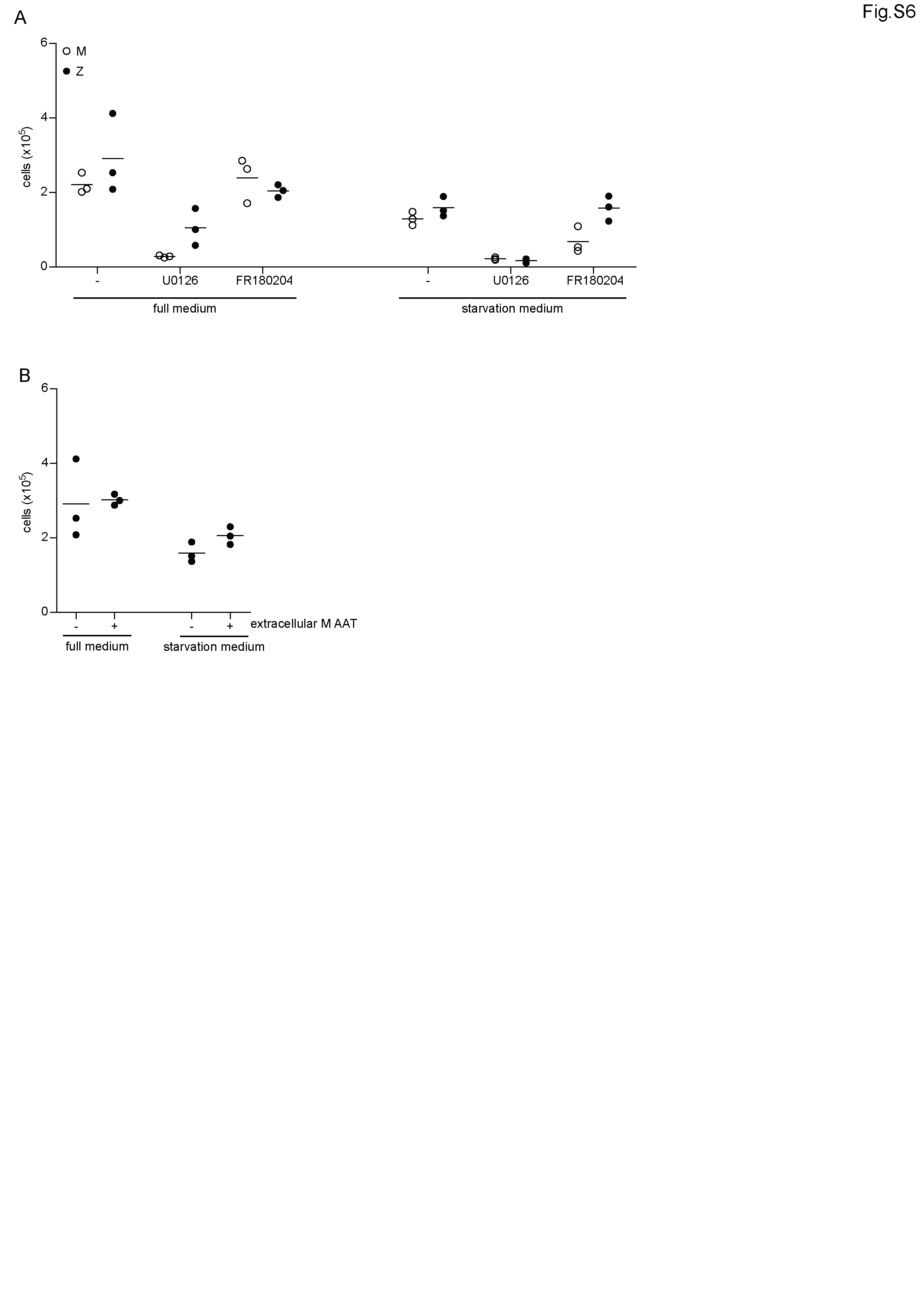

Supplement: Supplementary Data [file supp_ddt487_ddt487supp_fig6.tif]

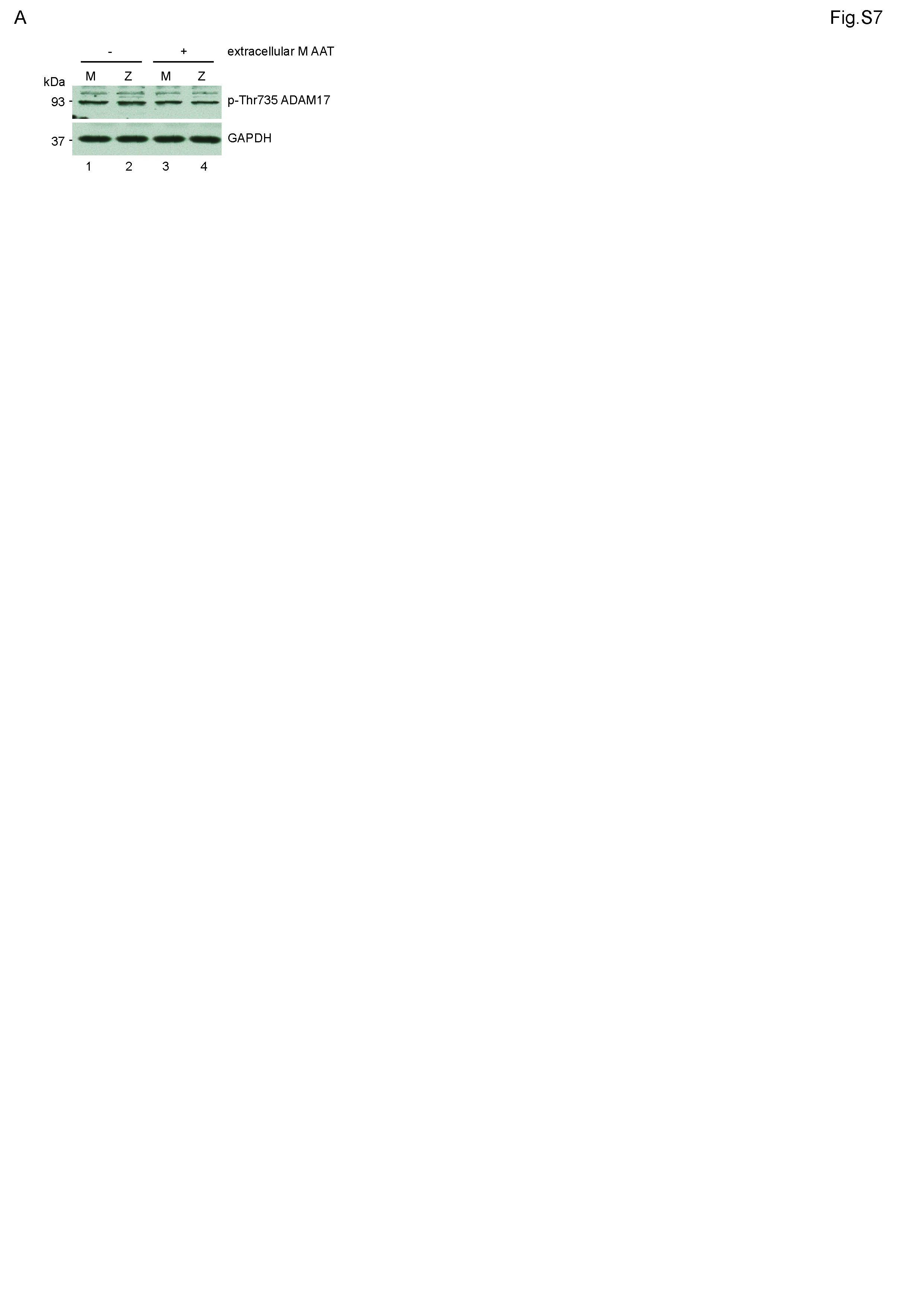

Supplement: Supplementary Data [file supp_ddt487_ddt487supp_fig7.tif]

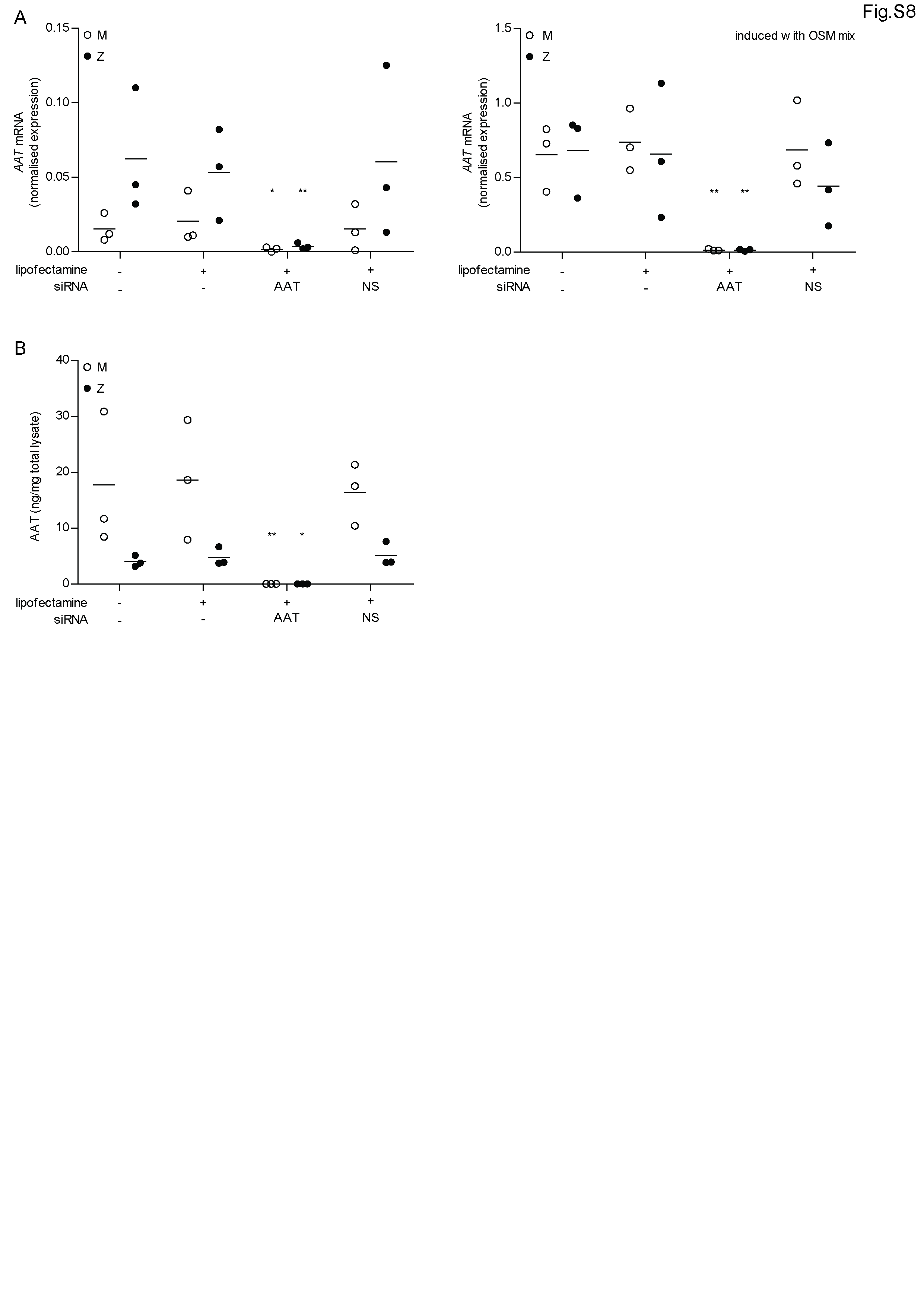

Supplement: Supplementary Data [file supp_ddt487_ddt487supp_fig8.tif]
